# New side-view imaging technique for observing posterior chamber structures during cataract surgery in porcine eyes

**DOI:** 10.1186/1471-2415-13-47

**Published:** 2013-09-23

**Authors:** Yoshitaka Tasaka, Noriyoshi Minami, Takashi Suzuki, Shiro Kawasaki, Xiaodong Zheng, Atsushi Shiraishi, Toshihiko Uno, Kensaku Miyake, Yuichi Ohashi

**Affiliations:** 1 Department of Ophthalmology, Medicine of Sensory Function, Ehime University Graduate School of Medicine, Shitsukawa, Toon, Ehime 791-0295, Japan; 2 Department of Ophthalmology, Ehime Prefectural Central Hospital, Kasugamachi 83, Matsuyama, Ehime 790-0024, Japan; 3 Minami Eye Clinic, Shimoshiromotomachi 1394-1, Hitoyoshi, Kumamoto 868-0015, Japan; 4 Shohzankai Medical Foundation, Miyake Eye Hospital, Ozone 3-15-68, Nagoya Kita-ku, Aich 462-0825, Japan

**Keywords:** Side-view imaging, Posterior chamber structures, Cataract surgery, Porcine eyes

## Abstract

**Background:**

To develop a side-view imaging technique for observing the dynamic behavior of posterior chamber structures (PCSs) in porcine eyes which mimics closed-eye cataract surgery in humans.

**Methods:**

Enucleated porcine eyes were placed into liquid nitrogen for 5 seconds and immediately bisected at about a 45-degree angle to the equatorial plane. The anterior portion was attached firmly to a glass slide with superglue and sprinkled with wheat flour. Phacoemulsification and aspiration (PEA) was performed as in humans on 10 consecutive porcine eyes. The movements of the PCSs were monitored through the glass slide with a high-resolution video camera set below the cut surface of the eye. The intraocular pressure (IOP) was monitored during the surgery. The highest IOP, operation time, and volume of irrigation fluid of 10 whole eyes were compared to that obtained from the bisected eyes glued to a glass slide. In a second set of experiments, the strength of the seal between the bisected eye and the glass slide was tested in three sets of eyes: 1) frozen eye fixed with superglue with wheat flour for 3 min; 2) frozen eye fixed with superglue for 3 min; and 3) non-frozen eye fixed with superglue for 30 min. The highest IOP that led to a disruption of the seal was compared among the three groups.

**Results:**

PEA was successfully performed on 9 of 10 (90%) eyes with the movements of the PCSs clearly observed. The average maximum intraocular pressure of the 9 bisected eyes was 55.8 ± 4.7 mmHg and that for the 10 unbisected eyes was 55.3 ± 5.0 mmHg (*P* = 0.650). The frozen eye fixed with superglue in combination with wheat flour (Group 1) had the strongest sealing strength with an average IOP at the breaking point of 117.3 ± 36.2 mmHg.

**Conclusions:**

Our side-view imaging technique can be used to evaluate the changes of the PCSs during intraocular surgery and for surgical training of new residents.

## Background

Over the past decade, phacoemulsification and aspiration (PEA) cataract surgery has become a safer and less invasive surgical procedure due to advances in surgical instruments and technology. To further minimize the intraoperative complications, it is necessary to understand how each surgical step might influence the intraocular structures especially the posterior chamber structures (PCSs), e.g., ciliary body, zonular fibers, anterior hyaloid membrane (AHM), and peripheral lens capsule. Because these structures are not visible to the surgeon during surgery, it is difficult to obtain information on their behavior during surgery. This is important because once these tissues are iatrogenically injured, a variety of complications can ensue. Therefore, new techniques to observe the behavior of the PCSs during surgery are necessary for better assessments of the surgery-related pathological changes.

The Miyake-Apple technique, originally developed in 1985 [1] and subsequently modified by Apple and Davis et al in 1990 [2], used postmortem human eyes and the preparation permitted real-time assessments of the intraoperative movements of the PCSs especially during the fixation of the intraocular lens. These methods have been widely used as research and educational tools to improve the quality and safety of PEA and cataract surgery.

However, the Miyake-Apple technique has some limitations. It enables the surgeon to observe only the lens and ciliary-zonular complex from the back of the eye, and this has limited the information on the behavior of the PCSs. In 1992, Assia and Apple developed an uveoscleral window technique in enucleated human eyes in which the lens and other PCSs could be seen by removing parts of the cornea, sclera, and ciliary body [3,4]. However, the behavior of PCSs could not be observed in closed-eye conditions by this method. In addition, a monitoring of the intraocular pressure (IOP) during the surgery was not possible.

To overcome these limitations, we have developed a new technique for examining the PCSs in postmortem porcine eyes. We were able to observe and video record the behavior of the PCSs continuously under closed-eye conditions as in human cataract surgery.

## Methods

### Preparation of porcine eyes

**Figure 1:**
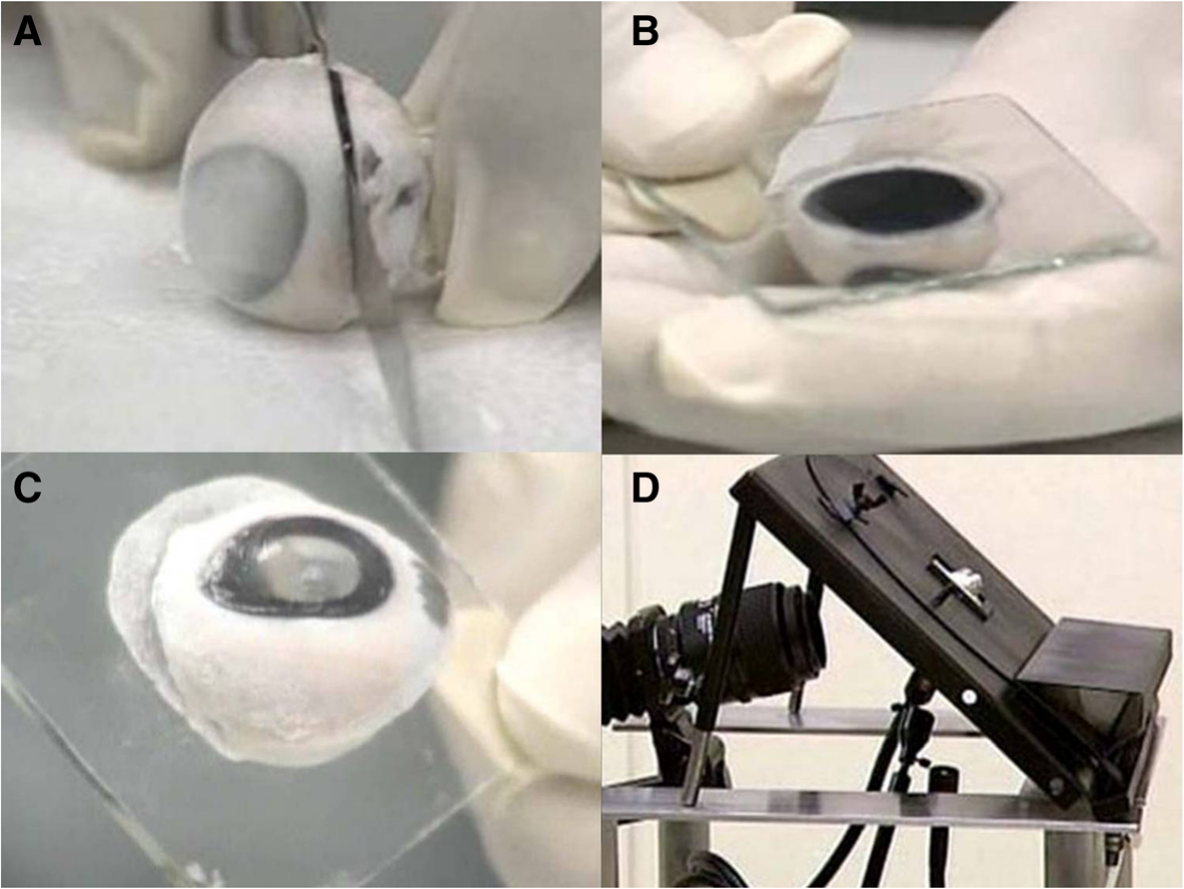
**Preparation of an enucleated porcine eye and side-view imaging technique. A**, The eye was frozen and transected at an angle of about 45 degrees to the equatorial plane with a utility knife. **B** and **C**, The bisected eye is fixed to a glass slide with a ring of superglue covered with wheat flour. The eye was left at room temperature for 3 minutes and a tight seal developed between the bisected eye and glass slide. **D**, Photograph of the equipment used for side-view cinematography.

Porcine eyes were obtained from a local abattoir and were stored at 4°C until used. All eyes were used within 10 hours of enucleation. Each globe was carefully inspected and eyes with lacerations or perforations were excluded. Eyes were dipped into liquid nitrogen for 5 seconds while holding the cut end of the optic nerve with forceps. This made the outer surface of the globe firm and enabled us to bisect the globe without altering its shape. The globe was bisected with a sharp utility knife at a 45-degree to the equatorial plane (Figure 1A). The anterior portion was used. All experimental protocols were approved by the Ethical Committee of Ehime University and comply with the ARVO Statement for the Use of Animals in Ophthalmic and Visual Research.

Then, approximately 0.2 grams of superglue (Aron alpha®, Toagosei, Japan) was applied in a circular pattern to a 50 mm × 50 mm pre-cleaned glass slide (Sharp cut filter® L37, HOYA Candeo Optronics, Japan). The glass slide was placed on the scleral rim taking care to avoid trapping air bubble (Figure 1B). After turning the slide over, 0.5 gram of wheat flour (Nissin Yuki®, Nissin Flour Milling Inc., Japan) was sprinkled onto the superglue as a bulking agent to promote solidification and to act as a filler of the interspaces. The eyes were left at room temperature for 3 minutes and a tight seal of the bisected eye to the glass slide developed.

### Side-view imaging technique

The bisected eye glued to the glass slide was placed on a surgical table specially designed for this technique (Figure 1C and D). The table was inclined at 45° so that the corneal surface of the glued eye faced directly upward. A video camera with a zoom lens (Lens: AF Micro-Nikon 105 mm f/2.80, Nikon, Japan; Attachment: BELLOWS PB-6, Nikon, Japan; Camera: DSR-1, SONY, Japan) was set beneath the surgical table and the camera lens was focused on the PCSs. The posterior surface of the iris, posterior chamber, ciliary body, zonular fibers, and the equator of the lens were in focus. The surgeon’s view camera was also connected and the images were video recorded simultaneously with the side-view images (BETACAM, SONY, Japan; Figure 1D).

### Measurement of intraocular pressure (IOP)

The IOP of the operated eye was monitored continuously as described in detail [5]. In brief, a 2.4-mm corneal incision was made 1.5 mm central to the limbus at the 9-o’clock position. Then a 16-gauge needle attached to a pressure sensor (DI-151RS; DATAQ Instruments, Inc, Akron, Ohio, USA) was inserted into the anterior chamber (AC). Care was taken to avoid any leakage of the infusion fluid from the 16-gauge needle.

### Phacoemulsification and aspiration procedure

After making a 2.8-mm corneal incision at the 12-o’clock position and a side port at the 2-o’clock position, the AC was filled with Healon® (AMO, USA). Then, a 5.5 to 6.0 mm continuous curvilinear capsulorrhexis was performed using capsulorrhexis forceps. The IOP was monitored through a 16-gauge needle attached to a pressure sensor in the AC. Hydrodissection was then performed (Nagahara cannula; ASICO Llc, Westmont, Illinois). The contents of the lens were emulsified with the bottle height at 75 cm, flow volume 26 ml/min, and aspiration rate 120 mmHg. The emulsified lens materials were removed by a Phacoemulsifier (Universal®, Alcon, USA) with a specially designed hook (MINAMI M-HOOK®, INAMI, Japan). Finally, the AC and the capsular bag were filled with Healon®, and an intraocular lens (AN6K®, Kowa Pharmaceutical, Japan) was implanted in the capsular bag using a lens forceps.

### Evaluation of sealing strength of bisected porcine Eye glued to glass slide

For our technique, it was essential that the bisected eyes glued to the glass slide be able to withstand fluctuations in the IOP during the surgery. To determine which sealing method was the strongest, we assessed the three different methods of fixation. In Group 1, frozen eyes were bisected and fixed to the glass with superglue and wheat flour. In Group 2, the eyes were prepared in the same way except that wheat flour was not applied. In Group 3, non-frozen eyes were bisected and fixed to the glass slide with superglue alone and left undisturbed for about 30 minutes, the Miyake-view procedure. A sharp 27-gauge needle attached to a 5-ml disposable syringe and attached to a pressure sensor was inserted into the AC at the 12-o’clock position. Then balanced saline solution (BSS) was slowly injected into the AC to increase IOP until the eye-slide glass seal was broken. The maximal IOP at the breaking point was recorded. Ten eyes from each group were tested.

In another set of experiments, bisected eyes were compared to whole eyes. Ten eyes each were tested for the changes of IOP during each surgical step of PEA, and the highest IOP, operation time, and volume of irrigation fluid used were compared.

### Statistical analyses

All data are expressed as the means ± standard deviations (SDs). Comparisons of the IOP among the groups tested for sealing strength of bisected eye glued to glass slide were evaluated by Tukey-Kramer’s test. Comparisons of the IOP between bisected eye and whole eye group tested during PEA were evaluated by Mann-Whitney test. A probability level of *P* < 0.05 was considered statistically significant. Data were analyzed with the JMP version 8.0 for Windows statistical software (SAS Japan Inc., Tokyo, Japan).

## Results

### Evaluation of sealing strength of bisected eye glued to glass slide

**Figure 2:**
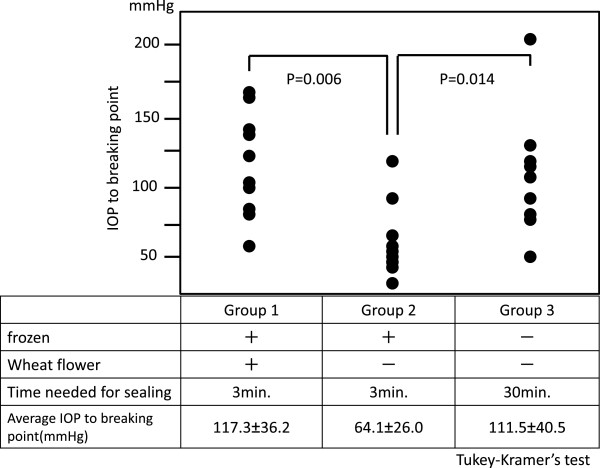
**IOP values corresponding to loss of integrity of the eye-slide glass seal.** The eye-glass slide seal was broken at a significantly lower IOP in frozen eyes with superglue and no wheat flour set for 3 minutes (Group 2) than that in Group 1 (*P* = 0.006) and Group 3 (*P* = 0.014, Tukey-Kramer’s test). There was no significant difference in the IOP at the breaking point between Groups 1 and 3 (*P* = 0.926, Tukey-Kramer’s test).

The average breaking pressure of the seal was 117.3 ± 36.2 mmHg in Group 1 (Figure 2), 64.1 ± 26.0 mmHg in Group 2, and 111.5 ± 40.5 mmHg in Group 3. The breaking pressure in Group 2 was significantly lower than that in Group 1 (*P* = 0.006) and Group 3 (*P* = 0.014, Tukey-Kramer’s test). The breaking pressure in Group 1 was not significantly different from that of Group 3 (*P* = 0.926, Tukey-Kramer’s test). Because the preparation of the bisected eyes with both superglue and wheat flour (Group 1) was rapid and effective, and because the eyes could tolerate the fluctuations in the IOP well, this method was used in all further experiments.

### IOP changes during PEA

**Figure 3:**
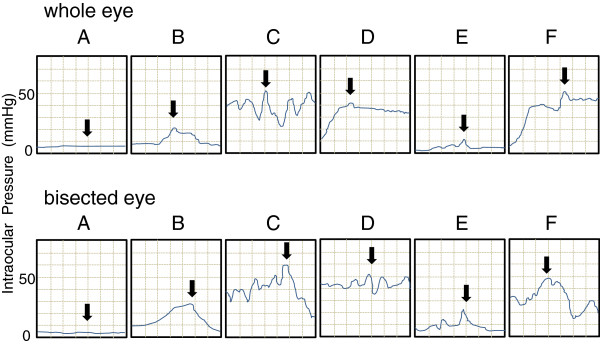
**IOP fluctuations during surgery. A**, After setting the pressure sensor, **B**, during hydrodissection; **C**, during ultrasound sonication (US); **D**, during irrigation/aspiration (I/A); **E**, during IOL insertion; **F**, during OVD aspiration. Top: Whole eye, A = 5.76 mmHg; B = 21.82 mmHg; C = 52.24 mmHg; D = 41.82 mmHg; E = 11.67 mmHg; and F = 52.01 mmHg. Bottom: Bisected eye; A = 4.85 mmHg; B = 28.03 mmHg; C = 59.70 mmHg; D = 52.27 mmHg; E = 23.33 mmHg; and F = 49.55 mmHg. Black arrows indicate peak IOPs. (X axis: 1 graduation = 0.5 seconds; Y axis: 1 graduation = 10 mmHg).

The IOP was monitored during PEA, and the changes in the IOP in representative cases of the whole eye and a bisected eye are shown in Figure 3. The fluctuations of the IOPs were similar for both groups. Although the shapes of graphs are somewhat different, the peaks of IOPs are comparable for all surgical procedure; viz, A, after setting the pressure sensor; B, during hydrodissection; C, during ultrasound sonication; D, during irrigation/aspiration; E, during IOL insertion; and F, during OVD aspiration (Figure 3). A summary of the highest IOP, operation time, and volume of irrigation solution used for the two groups is shown in Table 1. For bisected eyes, 9 of 10 (90%) eyes remained attached to the glass slide throughout the surgical procedures. The seal was broken in only one eye, and it occurred during ultrasound sonication of the lens. The average maximum IOP was 55.3 ± 5.0 mmHg for the whole eye group, and 55.8 ± 4.7 mmHg for the bisected eye group (*P* = 0.650, Mann-Whitney test). There was no significant difference in the average volume of the irrigation solution between the two groups (*P* = 0.107). The mean operation time was significantly longer in the bisected eyes than that for whole eyes (*P* = 0.028).

**Table 1:** Comparisons of parameters in whole and bisected eyes

**Group**	**Highest IOP (mmHg)**	**Operation time (sec.)**	**Irrigation volume (ml)**
Whole eye N=10	55.3±5.0	448.4±33.4	40.5±9.8
Bisected eye N=9	55.8±4.7	497.6±57.3	54.4±21.9
P value	0.650	0.028	0.107

IOP was monitored during PEA in two groups of porcine eyes (N=10 for each group). One group consisted of whole (unbisected) eyes, while the second group consisted of bisected eyes. The bisected eyes were glued to the glass slide with superglue and wheat flour. Among the bisected eyes, 9 of 10 (90%) eyes remained fixed to the slide throughout the procedure. The eye-glass slide seal was broken in only one case during ultrasound sonication of the lens. There was no significant difference between the two groups with respect to the maximum average IOP or the mean volume of the irrigation solution (Mann-Whitney test). The procedure time was significantly longer in bisected eyes than that of whole eyes (*P* = 0.028 Mann-Whitney test).

### Observations of PCSs with side-view technique

The movements of the PCSs were clearly visible with our side-view technique. The movements of the surgical instruments during PEA were also clearly seen. For example, during the removal of the nucleus, we were able to observe the insertion of the MINMI M-hook® deep into the capsular bag which facilitated the removal of the lens content. The implantation of the intraocular lens could also be seen in detail. Significant deformation of the zonular fibers indicated that the PCSs were under tension during these surgical maneuvers (Additional file 1).

Another advantage of this side-view imaging technique was that it allowed us to watch the flow of the irrigation solution through the bisected eye (Additional file 2). This was possible because the BSS irrigation fluid contained 1.0-μm fluorescein beads as described [5]. As shown in the first part of this video file, the fluorescein beads can be seen to be trapped in the zonular fibers (Additional file 2, Normal). This was probably due to the elevated IOP during the surgical procedures. During hydrodissection in some eyes, the space between the zonular fibers and anterior hyaloid membrane around the equatorial region of the lens increased, and numerous fluorescein beads were seen to scatter into the vitreous cavity through a tear in the anterior hyaloid membrane (AHM) around Wieger’s ligament (Additional file 2, AHT).

## Discussion

Damages to structures in the anterior chamber and central part of the posterior capsule can be clearly observed during intraocular surgery. However, it is difficult to see the damages in the posterior chamber, e.g., tearing of the zonular fibers [6,7]. Even if the stress on the zonules is transient, zonular weakness can lead to serious intra- or postoperative complications including zonular dialysis and vitreous prolapse. Although earlier reports have addressed the importance of the surgical tension on the zonular fibers and their surrounding tissues [8], the resilience and movements of the zonular fibers and AHM have not been well documented. One reason for this is the inability to view of the PCSs in a closed-eye surgical condition.

Our results showed that a side-view imaging system can provide useful information on the movements of the zonular fibers, the lens capsule, the ciliary body, and the AHM under a variety of surgical conditions during cataract surgery. Our technique is based on the elements of the Miyake-Apple posterior view and that of Assia and Apple side-view analysis of the lens [1]–[4]. However, our method has several advantages over these two methods.

First, cataract surgery can be successfully performed in a closed-eye surgery condition that mimics the surgical situation in human eyes. Khng et al. reported that the IOP was around 60-100 mmHg when the bottle height was at 130-150 cm during standard cataract phacoemulsification in cadaver eyes [9]. Our study showed that eyes prepared for side-view imaging system tolerated IOP elevations well and withstood pressure at a similar level of that of the whole eye for the tested period (Figure 3, Table 1). It is important that the side-view imaging technique with bisected eyes represent a practical surgical procedure in terms of having comparable values of the peak IOP as those of real “whole eye surgery”. In addition, the surgery in the bisected eyes was satisfactory in terms of the operation time and volume of irrigation fluid solution used (Table 1). The average IOP at the breaking point (117.3 ± 36.2 mmHg) was much higher than the highest IOP measured during surgery for the bisected eyes and the whole eyes. At a 75 cm bottle height, there was no difference between the bisected eyes and the whole eyes.

Another advantage is that bisected eyes can be readily prepared by using our technique of superglue in combination of wheat flour. Our data showed that application of wheat flour was especially helpful in creating a secure sealing of the eye to the glass slide. The mechanism for this is not fully understood, however we suggest that the flour may act as a bulking agent to promote solidification and act as a filler of the interspaces. In addition, components in the wheat flour might facilitate or accelerate the polymerization of the cyanoacrylate in the glue. Therefore, an enhanced strength of sealing of the scleral rim onto the slide glass was achieved in a short time.

Our side-view imaging technique was used to monitor the behavior of the PCSs and movements of surgical instruments during cataract surgery. With this technique, surgeons should be able to perform PEA while simultaneously watching the movements of the surgical instruments such as the phaco-tip and nucleus chopper. Therefore, surgeons can easily confirm the proper depth of the surgical field as well as verify the correct maneuver of the surgical instruments. This side-view imaging technique should be able to be used for training residents on intraocular surgery or on improving surgical instruments or techniques for intraocular surgery.

We have reported on the benefits of using fluorescein beads in the irrigation solution during surgery [5]. The size of fluorescein beads is 1.0-μm in diameter, which is similar to that of bacteria. This method is particularly useful for tracing the flow of irrigation fluids during surgical procedures. We have demonstrated that as the IOP increases, the posterior chamber-anterior hyaloid membrane barrier will undergo an elevation of pressure [5]. We confirmed that the fluorescein beads could be trapped by the dense network of zonular fibers during standard PEA (Additional file 2, Normal), indicating that the zonular fibers may act as an important barrier for the invasion of bacteria from entering the vitreous cavity. Furthermore, our method also clearly documented the formation of a AHM tear [10], and the tear could be a risk factor leading to endophthalmitis following uneventful surgery (Additional file 2, AHT).

There are some limitations in this study. First, although a brief freezing of the outer surface of the porcine eye with liquid nitrogen was helpful for rapid and effective bisection of the eye, one could argue that this treatment might have affected the anatomical integrity of the PCSs. However, our thermographic measurements showed that the temperature around the zonular fibers after dipping the eye into liquid nitrogen for 5 seconds was around 4°C, and scanning electron microscopy showed that the morphology of the PCSs was normal (data not shown). Thus, we conclude that the morphology of the PCSs was preserved during this procedure.

The second limitation was that we were unable to complete the standard PEA in 1 out of 10 processed eyes because of the loss of the seal of the scleral rim to the glass slide. This warrants future studies on ways to obtain stronger sealing for this side-view imaging technique.

## Conclusion

In conclusion, our side-view imaging technique can be a useful method of monitoring the movements of PCSs and the movement of surgical instrument during cataract surgery. It can be used for surgical training or evaluation of surgical procedures.

## Competing interests

The authors declare that they have no competing interests.

## Authors’ contributions

YT, NM and XZ: Conception and design, acquisition, analysis and interpretation of data, drafting of manuscript, administrative and technical support. YT, TS, SK, XZ and AS: Technical support and analysis and interpretation of data. TU, KM, XZ and YO: Supervision. Read and approved the final manuscript. All authors approved the manuscript for submission.

## Authors’ information

Yoshitaka Tasaka, and Noriyoshi Minami, contributed equally as co-first Authors.

## Pre-publication history

The pre-publication history for this paper can be accessed here:

http://www.biomedcentral.com/1471-2415/13/47/prepub

## Supplementary Material

Additional file 1 **Side-viewing technique during phacoemulsification and aspiration.** Side-view technique shows images of the PCSs and the movement of surgical instruments during PEA and insertion of the intraocular lens in an enucleated porcine eye. The zonular fibers, lens capsule, surgical instruments, and insertion of the intraocular lens can be seen. The right lower part of a screen is the surgeon's view. Click here for file

Additional file 2 **Observation of the flow of the irrigation fluid.** The zonular fibers are stained by 1.0-μm fluorescein beads and the flow of the irrigation solution in the capsule can be seen. This shows an anterior hyaloid membrane tear (AHT). Click here for file
